# An HSV-2 based oncolytic virus can function as an attractant to guide migration of adoptively transferred T cells to tumor sites

**DOI:** 10.18632/oncotarget.2817

**Published:** 2014-11-25

**Authors:** Xinping Fu, Armando Rivera, Lihua Tao, Xiaoliu Zhang

**Affiliations:** ^1^ Department of Biology and Biochemistry and Center for Nuclear Receptors and Cell Signaling, University of Houston, Texas, USA; ^2^ Department of Biology and Biochemistry, University of Houston, Houston, Texas, USA

**Keywords:** oncolytic virus, adoptive T cell transfer, chemokines, T cell migration, antitumor immunity

## Abstract

Adoptive T-cell therapy has shown promises for cancer treatment. However, for treating solid tumors, there is a need for improving the ability of the adoptively transferred T cells to home to tumor sites. We explored the possibility of using an oncolytic virus derived from HSV-2, which can actively pull T effector cells to the site of infection, as a local attractant for migration of adoptively transferred T cells. Our data show that intratumoral administration of this virus can indeed attract active migration of the adoptively transferred T cells to the treated tumor. Moreover, once attracted to the tumor site by the virus, T cells persisted in there significantly longer than in mock-treated tumor. Chemokine profiling identified significant elevation of CXCL9 and CXCL10, as well as several other chemokines belonging to the inflammatory chemokine family in the virus-treated tumors. These chemokines initially guided the T-cell migration to and then maintained their persistence in the tumor site, leading to a significantly enhanced therapeutic effect. Our data suggests that this virotherapy may be combined with adoptive T-cell therapy to potentiate its therapeutic effect against solid tumors that are otherwise difficult to manage with the treatment alone.

## INTRODUCTION

Engineering T cells for adoptive transfer is an emerging immunotherapeutic approach that has shown significant promise in recent clinical trials for cancer treatment [1]. It was primarily developed to overcome host's immune tolerance towards tumor-associated antigens, which has proven difficult to break by the conventional immunotherapy of vaccination strategy. Genetic engineering of T cells is predominantly conducted by engrafting the cells, either with a cloned high affinity T cell receptor (TCR) or with a chimeric antigen receptor (CAR) that contains a single chain antibody (scFv) with strong binding affinity to a selected tumor-associated surface antigen. Thus, the engineered T cells usually have a higher affinity to the targeted tumor cells than the endogenously generated effector cells. Furthermore, T cells that are engineered by CAR engraftment have additional advantages compared to conventional T effector cells. For example, due to the nature of scFv-mediated antigen binding, recognition of CAR-engrafted T cells is non-MHC restricted and independent of antigen processing. This brings two desirable consequences for cancer immunotherapy. First, it widens the application of these cells to patients with different MHC haplotypes. Second, the nature of non-MHC restriction allows recognition and destruction of tumor cells with down-regulated expression of MHC and/or altered antigenic peptide processing—one of the most important mechanisms by which tumor cells escape conventional cancer immunotherapy [2]. Adoptive transfer of engineered T cells that target a variety of tumor-associated antigens has been reported [3]. Encouraging preclinical data have prompted a series of clinical trials that use adoptive transfer of T cells for the treatment of tumors of different tissue origins, including melanoma [4], lymphoma [5-7], neuroblastoma [8], and colorectal cancer [9]. Many of these trials have shown measurable responses and, in some cases, complete remission of the established tumors [7].

However, despite these encouraging progresses, adoptive T cell therapy seems to be less effective for solid tumors than on hematologic malignancies. One particular area that may need improvement for solid tumor treatment is the ability of the engineered T cells to home to tumor sites after they are adoptively transferred to cancer patients by the systemic route. Unlike T effector cells that are generated during viral or bacterial infection, engineered T cells lack a well-defined chemotactic axis to actively migrate to tumor tissues [10]. To destroy established tumors efficiently, these adoptively transferred T cells must traffic to and infiltrate the tumor tissue. It is conceived that arming the engineered T cells with such a capability would greatly improve their therapeutic outcome.

Recent studies have shown that regional inoculation of type II herpes simplex virus (HSV-2) can actively “pull” both total and virus-specific effector T cells towards the infected area [11]. Apparently, this is due to the significant elevation of chemokine (C-X-C motif) ligand 9 (CXCL9) and CXCL10 expression, which was induced primarily by interferon-γ and, to a lesser extent, by type I interferon secretion at local tissues in response to HSV-2 infection. Subsequently, these chemokines attract the migration of effector CD8^+^ T cells to the infected tissue via the chemokine receptor CXCR3 [11]. CXCR3 is absent on naïve T cells, but it is rapidly upregulated following activation and remains highly expressed on type-1 helper (T_H_1)-type CD4^+^ T cells, effector CD8^+^ T cells [12]. As such, T effector cells strongly respond to the chemoattractant effect associated with HSV-2 infection, by actively migrating to the site of infection. Based on this finding, these investigators have recently developed a “prime and pull” strategy to deliberately mobilize activated T cells to the vaginal area for the purpose of preventing primary and recurrent HSV-2 infection [13].

We have developed an HSV-2-based oncolytic virus that can selectively lyse tumor cells without harming normal cells [14]. Designated FusOn-H2, it has potent antitumor activity against a variety of tumor types, and it acts either by direct oncolytic effect or by inducing host's antitumor immunity [14-19]. Here we conducted experiments to test the ability of this HSV-2-based oncolytic virus to serve as an attractant to guide engineered T cells to the tumor site during virotherapy. Our data show that intratumoral administration of FusOn-H2 can indeed attract active migration of the adoptively transferred T cells to the treated tumor. Moreover, once attracted to the tumor site by the virus, T cells persisted in there significantly longer than if they arrived in the mock-treated tumor without guidance. Chemokine profiling identified significant elevation of CXCL9 and CXCL10, as well as several other chemokines that belong to the inflammatory chemokine family. Together they initially guided the T-cell migration to and then maintained their persistence in the tumor site, leading to a significantly enhanced therapeutic effect. Thus, our data suggests that oncolytic HSV-2-based virotherapy may be combined with adoptive transfer of engineered T cells for efficacious treatment of solid tumors that are difficult to manage with the treatment alone. Both adoptive T-cell transfer and virotherapy have shown promises in phase III clinical trials as new cancer treatment modalities. As such, this combinatorial strategy could be feasibly translated into clinical application.

## RESULTS

### FusOn-H2 enhances migration of marked adoptively transferred T cells to the tumor site

**Fig.1:**
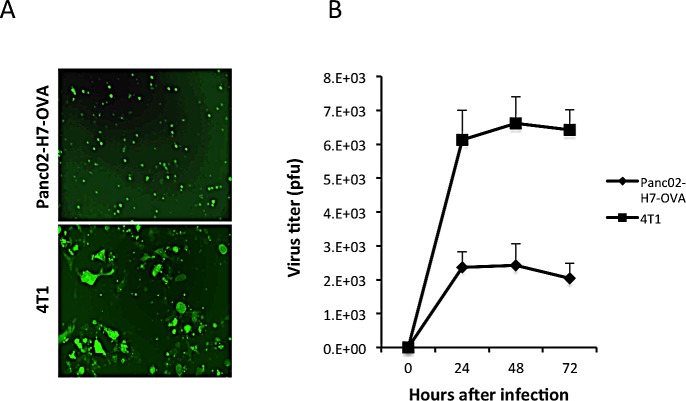
Comparison of permissiveness of Panc02-H7-OVA and 4T1 cells to FusOn-H2 A. Cells were infected with FusOn-H2 at 5 pfu/cell and micrographs were taken 24 h after infection. Shown is one typical field from each of the cells infected with the virus. Original magnification: 20X. B. Cells were infected with FusOn-H2 at 1 pfu/cell for 1 h. Then cells were harvested at the indicated time and the virus titer was determined by plaque assay of cell lysates on Vero cells.

Previous studies by Nakanishi et al showed that local administration of HSV-2 can “pull” engineered T cells that recognize a defined virus antigenic peptide towards the infection site [11]. To determine if the HSV-2 derived oncolytic virus, FusOn-H2, possesses a similar capability to pull tumor-specific T cells to the tumor site following virotherapy, we conducted an *in vivo* experiment using an OVA-expression tumor model in combination with splenocytes (OT-I cells) harvested from OT-I TCR transgenic mice [20]. The OVA-expressing tumor cell line, Panc02-H7-OVA, was established from the highly metastatic Panc02-H7 murine pancreatic adenocarcinoma cell [21]. We initially determined the permissiveness of Panc02-H7-OVA to FusOn-H2 and compared it with that of 4T1 cells, a murine mammary tumor line that we had used extensively in our previous oncolytic HSV studies [17, 22]. As FusOn-H2 contains the gene encoding for green fluorescent protein (GFP), its infectivity can be conveniently detected under a fluorescent microscope. The results in Fig.1 show that, although Panc02-H7-OVA cells can be infected by FusOn-H2, they are significantly less permissive than 4T1 cells to the virus infectivity (Fig.1a) and replication (Fig.1b). Additionally, FusOn-H2 seems to have lost its fusogenic phenotype in Panc02-H7-OVA cells, as the infected 4T1 cells predominately present as syncytia while infected Panc02-H7-OVA cells appear mainly as single individual GFP^+^ cells (Fig.1a). Low permissiveness and lack of syncytial formation are considered as an advantage for the subsequent *in vivo* experiments, as the oncolytic effect from FusOn-H2 would be limited and the majority of the treated tumor would survive so that the attractant effect from the virus could be fully evaluated.

**Fig.2:**
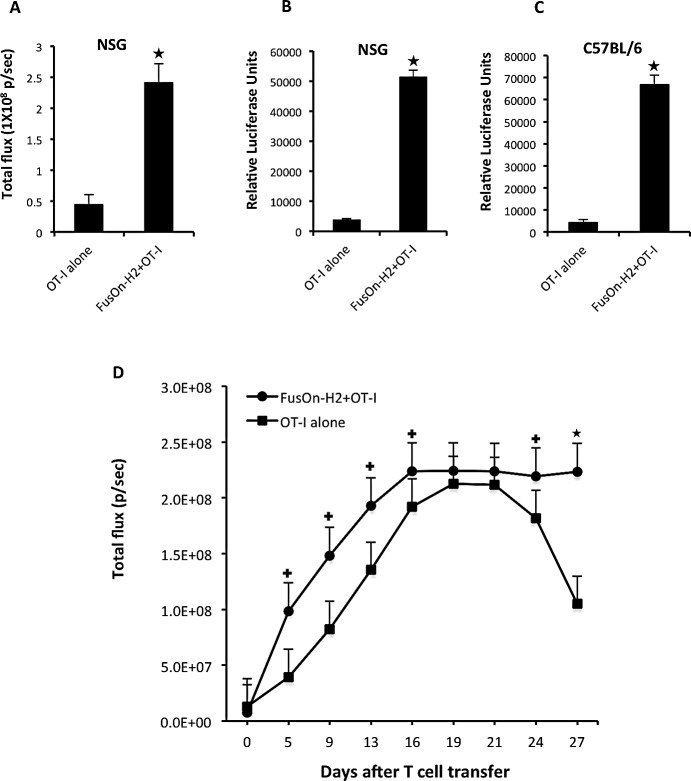
Attractant effect of FusOn-H2 on OT-I cell migration to tumor site and the subsequent in situ expansion of OT-I cells Murine pancreatic tumors were established by implanting Panc02-H7-OVA cells in the right flank of both immunodeficient NSG mice (A, B and D) and syngeneic C57BL/6 (C). Once tumors reached the approximate size of 5 mm in diameter, mice received intratumoral injections of either PBS or 5×10^6^ pfu of FusOn-H2. Twenty-four h later, all mice received an intravenous infusion of 2×10^6^ OT-I cells that had been transduced with a luciferase-containing retroviral vector. The abundance of OT-I cells that had migrated to tumor sites was determined either by IVIS imaging (A and D) and/or luciferase assay (B and C). A and B were from the same animals. The IVIS image in A was taken at day 28 (after adoptive cell transfer) immediately before the animals were euthanized to collect tumor for luciferase assay. +p<0.05, *p<0.01 as compared with OT-I alone.

To facilitate *in vivo* tracking, the OT-I cells were transduced with a retrovirus containing *luciferase* gene forty-eight hours before adoptive transfer. Tumors were established subcutaneously on both the immunodeficient NSG mice and the immunocompetent syngeneic C57BL/6 mice with implantation of Panc02-H7-OVA cells, which are an OVA expressing cell line that was established from the highly metastatic Panc02-H7 murine pancreatic adenocarcinoma cell [21]. The main reason for including the immunodeficient NSG mouse in this experiment is because the immunodeficient nature with complete absence of T cells in NSG mice would allow easy and unambiguous characterization of the adoptively transferred OT-I cells. Once tumors reached the approximate size of 5 mm in diameter, they were either mock-treated or injected intratumorally with 1×10^7^ plaque-forming units (pfu) of FusOn-H2. Twenty-four hours later, all mice received an adoptive transfer of 2×10^6^ OT-I cells that had been transduced with a luciferase-containing retrovirus. NSG mice were imaged four days after adoptive cell transfer and the quantified image data was presented in Fig. 2a. On average, there was more than a six-fold increase of the photon flux in the tumors treated with FusOn-H2 than in the mock-treatment after adoptive transfer of OT-I cells transduced with luciferase-containing retrovirus. To corroborate the results deduced from photon flux and to more accurately quantitate OT-I cells that had homed to the tumor site, both NSG and C57BL/6 mice were sacrificed, and tumors were collected for direct measurement of luciferase activity. The results showed an almost 14-fold increase on the luciferase activity in tumors treated with FusOn-H2 as compared to mock-treatment in NSG mice (Fig.2b). As the imaging data in Fig.2a was obtained from the same mice, the results in Fig.2b thus indicate a good correlation between the accurate *in vitro* luciferase assay and the *in vivo* imaging estimation. *In vitro* luciferase assay on the syngeneic tumors obtained from C57BL/6 mice showed a 16-fold increase in activity when comparing FusOn-H2 to mock treatment, indicating that the virus produces similar attractant effect on OT-I cells in both tumor models. Together, these data show that local administration of FusOn-H2 can attract the active migration of tumor-specific T cells and possibly other components of splenocytes to the tumor site after the adoptive cell transfer.

### Characterization of OT-I cells migrated to the tumor site

To monitor the status of OT-I cells migrated to the tumor site, we regularly imaged NSG mice that had been treated the same way as described in Fig.2a, for an extended period of time. The quantitated image data is presented in Fig.2D. Initially there was a steady increase in the bioluminescent signal in tumors from both groups, indicating a tumor-specific T-cell expansion that was independent of virus infection. The bioluminescent signal reached the peak level at around 16-19 days after the adoptive transfer of OT-I cells. Then, the signal in the mock-treated tumor started to decline and the declination continued until day 28, which was the end of the experiment. In contrast, bioluminescent signal from the FusOn-H2-treated tumor maintained the peak level until the end of the experiment, indicating that FusOn-H2 treatment might have contributed to this persistence.

**Fig.3:**
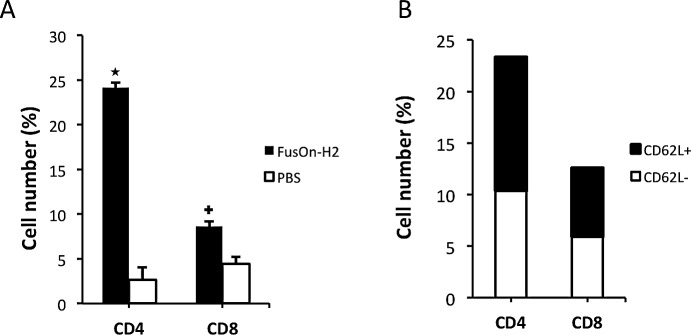
Characterization of T cell subsets of OT-I origin within tumors Tumors were explanted from mice used in Fig.2, which had been treated either with PBS (mock) or FusOn-H2 before adoptive transfer of OT-I cells. After the Histopaque density gradient separation of cell suspension was prepared from the harvested tumors, the layer containing lymphocytes and monocytes was collected and stained for CD4 and CD8 (A) or CD4/CD62L and CD8/CD62L (B), respectively. The data in B is from tumor treated with FusOn-H2. Due to the low number of CD4 and CD8 T cells that could be harvested from the tumor treated with PBS, the number of CD4/CD62L and CD8/CD62L subpopulations was insufficient for a reliable quantitative measurement by flow cytometry. The percentage represents the positively stained cells out of the total cells enriched within the layer. +p<0.05, ^*^p<0.01 as compared with PBS.

To dissect the composition of T-cell subsets of OT-I origin within the tumor tissues, tumors were harvested from mice described in Fig.2D after the termination of the experiment. After Histopaque density gradient separation, the layer enriched with lymphocytes and monocytes was collected and stained for CD4 and CD8, respectively. Fig.3a shows the flow cytometry analysis of one representative sample from each treatment group. In accordance with the bioluminescent imaging data in Fig.2D, the percentage of both CD4^+^ and CD8^+^ T cells was significantly higher than that from mock-treated tumors. This difference was particularly noticeable for CD4^+^ cells; the percentage of CD4^+^ cells in FusOn-H2 treated tumors was almost 10-fold higher than that in the mock-treated tumors (24.9% vs. 2.66%). The CD8^+^ cells showed a 2-fold difference between the two groups (7.69% vs. 4.64%). The rest of the cells that were not positively stained for either CD4 or CD8 are most likely tumor associated macrophages. We further analyzed the harvested OT-I cells from FusOn-H2-treated mice for memory phenotype by staining them for CD4/CD62L or CD8/CD62L. CD62L/L-selectin is a marker for central memory T cells [23]. The results show that a significant percentage of CD4^+^ and CD8^+^ cells was stained positive for CD62L (Fig.3b). Together, these data suggest that FusOn-H2 can initially guide the migration of adoptively transferred T cells towards the treated tumor. It can then help maintain their persistence once they have arrived and proliferated at the tumor site, with the majority of persistent T cells as CD4^+^ subset. Many of the T cells that persist eventually convert to memory phenotype.

### Chemokine profile in FusOn-H2 treated tumors that attract migration of OT-I cells

**Fig.4:**
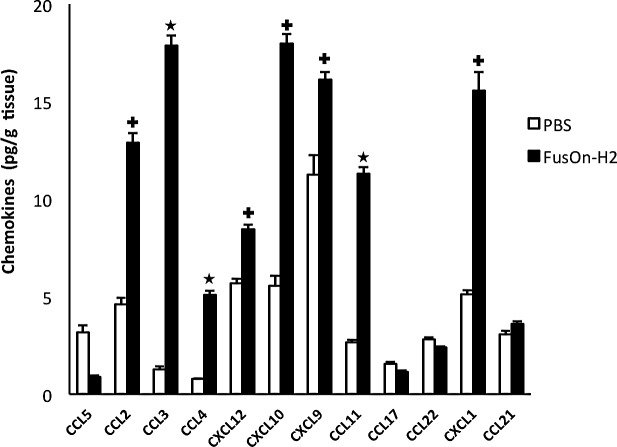
Comparison of chemokine profile between mock and FusOn-H2 treated tumors Panc02-H7-OVA tumors were established in NSG mice and treated with PBS or 1×10^7^ pfu of FusOn-H2. Tumors were explanted two days after treatment, and the tumor lysates were used for chemokine measurement using the Qiagen's Multi-Analyte ELISArray kit. +p<0.05, ^*^p<0.01 as compared with PBS.

To determine the chemokine profile during treatment of tumors by FusOn-H2 that is associated with the guided migration of the adoptively transfer OT-I cells to tumor site, we established Panc02-H7-OVA tumor in NSG mice. Animals then received an intratumoral injection of either PBS (mock) or FusOn-H2 as described in Fig.2. Forty-eight hours later, tumors were explanted for quantitative measurement of a panel of 12 chemokines. The results show that a cluster of chemokines (including CXCL9, CXCL10, CXCL11) that have been reported by Nakanishi et al to be involved in the homing of HSV-2 specific T cells to the virus infection site [11], were also found to be significantly increased in FusOn-H2 treated tumors (Fig.4). However, the CXCL11 gene in C57BL/6 mouse strain, from which NSG mouse was developed, contains a point mutation and a single-base deletion that results in a reading frame shift. Hence, a non-functional CXCL11 protein product is produced [24]; only CXCL9 and CXCL10 are probably the functional chemokines in this cluster to act on and to attract OT-I cell migration to the treated tumors. The data also revealed significant elevation of several additional chemokines. Among them is the cluster that contains CCL2, CCL3 and CCL4. In fact, CCL3 is the highest elevated chemokine (more than 14-fold increase) among the 12 chemokines that had been assayed. CXCL1 is another chemokine that showed a significant increase after FusOn-H2 treatment. On the other hand, the levels of CCL17, CCL21 and CCL22 were not affected by FusOn-H2 infection, and CCL5 was actually reduced by the virus treatment.

**Fig.5:**
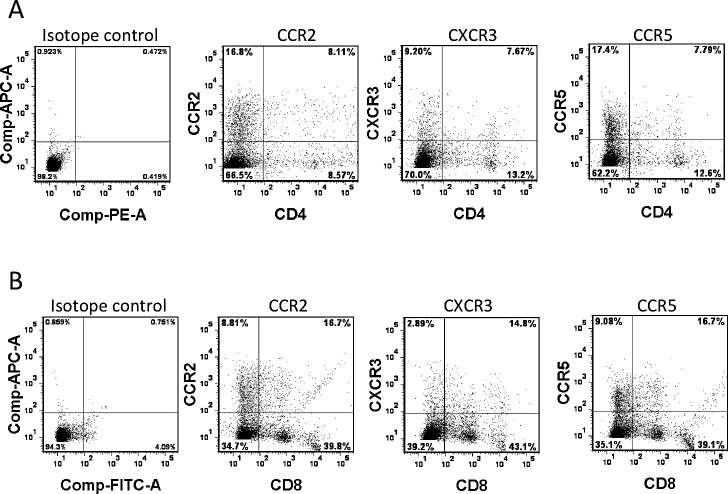
Chemokine receptor expression on OT-I cells Splenocytes were harvested from 6-week-old OT-I mice. After overnight culture with RPMI 1640 complete medium containing IL2, cells were doubly stained for chemokine receptors (CXCR2, CCR3 and CCR5) and either CD4 (a) or CD8 (b), or the isotope controls, and analyzed by flow cytometry.

CXCL9, CXCL10 and CXCL11 all bind to a common primary receptor, CXCR3, to chemoattract T lymphocyte migration [25]. CCL2, CCL3 and CCL4, on the other hand, bind to chemokine receptor CCR2 (CCL2) and CCR5 (CCL3 and CCL4), leading to the chemoattractant effect on monocytes, immature dendritic cells, natural killer cells and activated T cells [26, 27]. We thus doubly stained splenocytes harvested from OT-I mice for each of these three chemokine receptors, in combination with either CD4 or CD8. All three chemokine receptors were readily detectable at a significant percentage of both CD4^+^ (Fig.5a) and CD8^+^ (Fig.5b) cells. These results, together with the data in Fig.4, indicate that, in the context of tumors treated by FusOn-H2, more than one cluster of chemokines have been elevated. They act on their primary receptors to impact OT-I cell migration to and persistence in the tumor tissue.

### Guided migration of OT-I cells to the tumor sites by oncolytic HSV enhances therapeutic effect

**Fig.6:**
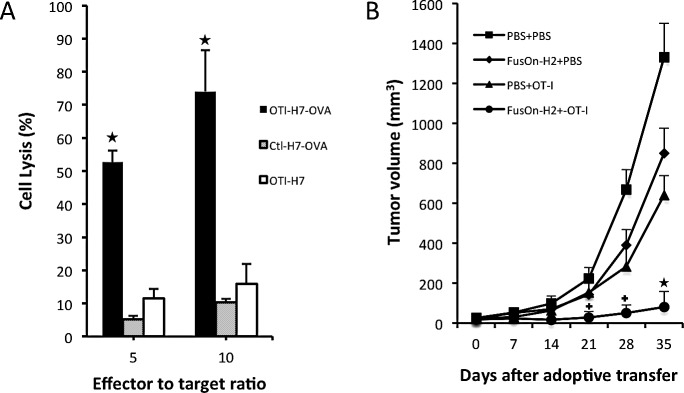
*In vitro* cytotoxicity of OT-I cells against Panc02-H7-OVA target cells and *in vivo* therapeutic evaluation on FusOn-H2 guided migration of OT-I cells A. Splenocytes were harvested from either OT-I mice (OTI) or wild type C57BL/6 mice (Ctl). After overnight culture with RPMI 1640 complete medium containing IL2 at the concentration of 300 U/ml, they were mixed with were either Panc02-H7-OVA (H7-OVA) or Panc02-H7 (H7) cells at the indicated ratio. The cytotoxicity was determined by cell viability measurement and calculated by the formula: the percentage of cell killing = (1-viable cell number with effector-cell/viable cell number in the control) X100. ^*^p<0.01 as compared with OT-I alone. B. Panc02-H7-ova tumors were established in the right flank. Mice received intratumoral injections of either PBS or 1×10^7^ pfu of FusOn-H2 two days before they were adoptively transferred with 2×10^6^ OT-I cells or PBS (n=5 mice per group). Tumor size was periodically measured and plotted against the time after treatment. +p<0.05, ^*^p<0.01 as compared with FusOn-H2 or OT-I alone.

Before the *in vivo* experiment was conducted to evaluate therapeutic efficacy, the cytotoxic activity of OT-I cells against Panc02-H7-OVA cancer cells was assessed *in vitro*. Several controls were included in this *in vitro* experiment to assert the killing specificity. Panc02-H7-OVA cells or the parental Panc02-H7 cells were mixed with OT-I cells at different effector-to-target (E:T) ratios. In another well, Panc02-H7-OVA cells were mixed with splenocytes harvested from wild type C57BL/6 mice. The cytotoxicity was subsequently determined as described in the Materials and Methods. The results show that, as expected, OT-I cells have strong cytotoxicity against Panc02-H7-OVA cells but not the parental cells that lack OVA antigen expression (Fig.6a). The splenocytes from C57BL/6 cells did not show significant cytotoxicity against Panc02-H7-OVA cells even at the higher E:T ratio of 10:1. Thus, these *in vitro* data confirm that Panc02-H7-OVA cells, which were established from the popular aggressive murine Panc02 tumor cells, can be efficiently recognized by OT-I cells for specific killing.

To investigate if FusOn-H2 mediated guidance of OT-I cell migration to the tumor site benefits the therapeutic efficacy, Panc02-H7-OVA tumors were established in NSG mice. Once tumors reached the approximate size of 5 mm in diameter, mice received intratumoral injections of either FusOn-H2 or PBS. This was followed by adoptive transfer of either OT-I cells or PBS by an intravenous route. Afterwards, tumor volume was monitored periodically and plotted (Fig.6b, the data on tumor growth of individual animals was presented as supplementary figure 1). FusOn-H2 alone showed little therapeutic effect against this murine tumor, as the Panc02-H7 cells are highly resistant to the lytic effect of HSV-derived oncolytic viruses such as FusOn-H2. Administration of OT-I cells alone showed a very moderate inhibitory effect on the tumor growth. The best result came from the combination treatment of local administration of FusOn-H2 followed by systemic delivery of OT-I cells, which resulted in a profound inhibition on the tumor growth. By the end of the experiment, two mice from this treatment group were tumor free, and the rest had tumors that were much smaller than in other groups. The two tumor-free mice were subsequently challenged with 1×10^6^ Panc02-H7-OVA tumor cells to the opposite flank. Tumor failed to grow on both animals, indicating that these animals were fully protected by the adoptively transferred OT-I cells that apparently underwent expansion and then persisted in the tumor.

## DISCUSSION

Development of an active mechanism to guide the adoptively transferred T cells to tumor sites is desirable for maximizing the therapeutic outcome of this treatment modality. Several strategies have been previously explored for this purpose. The most common strategy is to transduce tumor-specific T cells with a chemokine receptor. For example, it has been reported that expression of chemokine receptors CXCR2 and CCR2 by CAR modified T cells can facilitate their localization to the tumor site and hence antitumor immune responses [28, 29]. Co-expression of CCR4 and a chimeric antigen receptor targeting CD30 by T cells has also been shown to improve homing and antitumor effect in a Hodgkin tumor model [30]. Other studies have explored the possibility of using oncolytic vaccinia virus to express chemokines such as CCL5 and CCL19 to enhance the therapeutic effect by increasing T cell and dendritic cell infiltration into tumor tissues [31, 32].

Prompted by recent reports that HSV-2 infection can guide migration of virus-specific T effect cells to the site of virus infection [11, 13], we investigated the possibility of using a HSV-2-derived oncolytic virus as an attractant to actively guide migration of adoptively transferred T cells to the treated tumor without carrying any transgene. We chose to conduct this experiment in immunodeficient rather than wild type mice as we believed that the immunocompromized condition in the former is more clinically relevant. This is because cancer patients who receive adoptive T cell therapy usually need to undergo myeloablation or lymphodepletion in most clinical recipes. Our data showed that indeed OT-I cells, which are specific for OVA-expressing tumor cells, were positively pulled to Panc02-H7-OVA tumor when it was treated with the HSV-2-based oncolytic virus, FusOn-H2. Our data further showed that such a guided migration of OT-I cells led to a significantly improved therapeutic effect against a very aggrieve pancreatic cancer that otherwise responded poorly to ether virus treatment or the adoptive T cell transfer alone.

We believe our strategy of employing an oncolytic virus to guide the migration of tumor-specific T effector cells to the tumor site has several distinctive advantages over other methods. First, in addition to its ability to function as an attractant for adoptively transferred T cells, virotherapy can alter the tumor microenvironment in a fashion similar to that of natural virus infection of other organ tissues, which has been shown to promote T cell proliferation and persistence [11]. Indeed, our imaging data in Fig.2D showed that, after arriving in tumor sites, OT-I cells initially underwent a proliferation that was more profound in FusOn-H2-treated tumors than in the mock-treatment controls. The optical imaging data further showed that the expanded OT-I cells then survived and persisted in FusOn-H2-treated tumors significantly longer than in the mock-treated tumors. Interestingly, studies by Nakanishi et al have shown an initial recruitment and subsequent expansion of CD4^+^ T cells in the HSV-2 infected region and they conclude that these CD4^+^ T cells are required for the mobilization of antigen-specific CD8^+^ cells [11]. Our data showed a similar CD4^+^ cell expansion. Since these CD4^+^ cells are not supposed to react to OVA, their expansion was most likely due to the presence of FusOn-H2 infection. Second, unlike other methods reported in the literature, this strategy can be applied to guide the tumor-orientated migration of the endogenous tumor-specific T cells, which can be generated by virotherapy or other vaccination approaches. Third, as compared with the oncolytic vaccinia virus expressing chemokines [31, 32], our approach uses an oncolytic HSV-2 that does not need to carry any external transgene. As such, it has less safety concern.

Studies by Nakanishi et al demonstrate that CXCL9 and CXCL10, two major T cell chemoattractant chemokines, are mainly involved in “pulling” the migration of virus-specific T effector cells towards the infected vagina mucosa [11]. Our data show that indeed the release of these two chemokines was significantly induced in the FusOn-H2-treated tumors as compared with the mock treatment. The receptor for these two chemokines, CXCR3, was also detected in a significant percentage of OT-I cells. These suggest that CXCL9 and CXCL10 are also the major chemokines that guide the adoptively transferred OT-I cells to the treated tumor. However, several chemokines that belong to the inflammatory chemokine cluster were also significantly induced in tumors treated with FusOn-H2. These include CCL2 (aka monocyte chemotactic protein-1, MCP-1), CCL3 (aka macrophage inflammatory protein-1α, MIP-1α) and CCL4 (aka macrophage inflammatory protein-1β, MIP-1β). The receptors for these chemokines, CCR2 and CCR5, were also readily detectable on OT-I cells. The primary function of these chemokines is to recruit effector cells, including monocytes, granulocytes and effector T cells to the infection site [33]. As such, these chemokines may have partly contributed to the recruitment of OT-I cells to the FusOn-H2 treated tumors directly. The indirect recruitment effect on OT-I cells may come from interferon-γ (IFN-γ) released by these initially arrived effector cells. For example, Benencia et al have reported that intratumoral injection of a HSV-1-based oncolytic virus can stimulate monocytes and dendritic cells to secret IFN-γ [34]. Based on the report that CCR2 and CCR5 are highly expressed on helper T cells and that CCL3 can attract the migration of CD4^+^ T cells to the site of infection [35-37], we speculate that another consequence of the drastically induced CCL3 by FusOn-H2 is the recruitment of CD4^+^ T cells to the tumor site, which can then secret abundant IFN-γ. IFN-γ is essential as well as sufficient to induce secretion of CXCL9 and CXCL10 for subsequent recruitment of T effector cells as demonstrated by Nakanishi et al during natural HSV-2 infection [11]. The increased release of inflammatory chemokines, on the other hand, might have played a role in prolonging the persistence of OT-I cells in FusOn-H2 treated tumors. One possible mechanism for this effect is that they promote the recruited T cells to become memory phenotype, as demonstrated in Fig.3b. Indeed, it has been reported that CCL2 expression is associated with accumulation of an activated memory subset of T cells during tissue injury or infection [38], and studies by Castellino et al have shown that blockade of CCL3 and CCL4 can markedly reduce the ability of CD4^+^ T cells to promote memory OT-I (CD8^+^) generation [39].

When the concept of cancer virotherapy was originally conceived, it was assumed that the therapeutic effect would exclusively come from the virus-mediated oncolysis. However, it is becoming clear that antitumor immunity is frequently generated during virotherapy and it plays an important role in contributing to the overall antitumor activity. As a consequence, efforts have been made in arming oncolytic viruses with mechanisms to further potentiate their immunotherapeutic effect. For example, studies by several groups as well as from our own have shown that destruction of murine tumors by oncolytic viruses *in vivo* can generate measurable tumor-specific immunity in the syngeneic mouse models [17, 18, 22, 40-44]. Our own studies on oncolytic HSVs have shown that the killing mechanisms associated with virotherapy can dictate the magnitude of the antitumor immune response. For example, we found that tumor destruction by oncolytic HSVs with fusogenic property could induce a robust antitumor immune response even against weakly immunogenic tumors [17, 18, 22, 45]. In particular, a fusogenic oncolytic virus that we have constructed from HSV-2, the first of this kind and is designated FusOn-H2 [14], has a strong ability in inducing antitumor immunity in several syngeneic tumor models [17-19].

In summary, our data suggest that intratumoral administration of an HSV-2-based oncolytic virus can function as an attractant to guide adoptively transferred T effector cells to the tumor site for an enhanced antitumor effect. Both virotherapy and adoptive T cell transfer are currently at different phases of clinical trials (including one HSV-based oncolytic virus currently in Phase III clinical trial). As such, this combined strategy can be feasibly translated into clinical application in the near future.

## MATERIALS AND METHODS

### Cell lines and viruses

African green monkey kidney (Vero) cells were obtained from American Type Culture Collection (Rockville, MD, USA). Retroviral Packaging Cells (Plat-E) were from Cell Biolabs, Inc. (San Diego, CA). Panc02-H7 was kindly provided by Dr. Min Li (UT Health Science Center at Houston, TX). The Panc02-H7-OVA cell line was established by co-transfecting pIR-OVA plus pCMV-piggyBac plasmids, and selected with puromycin. 4T1 was kindly provided by Dr. Fred Miller (Michigan Cancer Foundation, Detroit, MI). The construction of the HSV-2-based oncolytic virus FusOn-H2 has been previously described [14].

### Antibodies and Reagents

APC anti-mouse CXCR3 and APC anti-mouse CCR5 antibodies were purchased from BioLegend (San Diego, CA). APC anti-mouse CCR2 was from R&D Systems, Inc. (Minneapolis, MN). FITC anti-mouse CD8, PE anti-mouse CD4 and anti-mouse CD16/32 antibodies were from BD Pharmingen (San Diego, CA). APC anti-mouse CD62L antibody was from BioLegend (San Diego, CA).

### *In vitro* assay of virus infection and replication in tumor cells

Panc02-H7-OVA and 4T1 cells seeded in 6-well plates in triplicates were infected with FusOn-H2 at 5 plaque-forming units (pfu) per cell for 1 h. Cells were washed three times with PBS before they were either harvested immediately or incubated for 24 h. Micrographs were taken under a fluorescent microscope immediately before harvest. Virus titer was determined by plaque assay.

### Preparation of OTI cells

OT-I cells were derived from spleens of C57BL/6-Tg (Ins2-TFRC/OVA) 296 Wehi/WehiJ transgenic mice that were purchased from the Jackson Laboratory (Bar Harbor, Maine). Splenocytes were harvested, filtered through 70 micron screeners and frozen at −20°C for later use.

### Transduction of OT-I cells with retroviral vectors

pRV-luc plasmid was co-transfected in Plate-E with the packaging plasmid, pCL-ECO, using lipofectamine 2000 (Invitrogen, Carlsbad, CA). Supernatants were collected 48 and 72 h later and were combined to generate stocks, which were then used to transduce OT-I cells. Briefly, single-cell suspensions of OT-I cells were activated with Concanavalin A (2 μg/ml; Sigma, St. Louis, MO) and mouse IL7 (1ng/ml; PeproTech, Rocky Hill, NJ) for 24 h. Cells were then transduced with pRV-luc retrovirus in non–tissue culture 24-well plates precoated with RetroNectin (Takara Bio. Inc., Shiga, Japan). The transduced OT-I cells were then cultured for 48 hours in fresh medium supplemented with hIL-2 (300 U/ml; NIH AIDS Reagent Program, Germantown, MD) to allow the cells to recover. They were then used directly for adoptive transfer.

### *In vitro* OT-I cytotoxicity assay

For *in vitro* OT-I cytotoxicity assay, Panc02-H7-OVA cells or the parent Panc02-H7 cells were seeded in 48 well plates. OT-I cells or splenocytes from wild type C57BL/6 mice were added to the wells at effector to target (E:T) ratios of either 5 or 10. The wells without added OT-I cells or splenocytes served as the controls. After 24 h incubation, OT-I cells or splenocytes were removed and the wells were gently rinsed with PBS two times. The cells that remained attached to the wells were trypsinized, stained with trypan blue for viability, and were counted under a microscope. Cytotoxicity was determined by the formula: the percentage of cell killing = (1- viable cell number with effector-cell/viable cell number in the control) x 100.

### *In vivo* tumor establishment and treatment

All animal experiments were approved by the University's Institutional Animal Care and Use Committee (IACUC). For establishing tumors, 1×10^6^ Panc02-H7-OVA cells were implanted into the right flank of either immunocompetent C57BL/6 mice or the immunodeficient NOD.Cg-*Prkdc^scid^ Il2rg^tm1Wjl^*/SzJ (NSG) mice (Jackson Laboratories, Bar Harbor, ME). Once tumors reached the approximate size of 5 mm in diameter, the animals were given a single intratumoral injection of either PBS or 1×10^7^ plaque forming unit (pfu) of FusOn-H2. For the animals receiving subsequent adoptive cell transfer, 5×10^6^ OT-I cells were given intravenously by the tail vein 24 h after virus injection. The tumor growth was monitored weekly by measuring two perpendicular tumor diameters with a caliper. Tumor volume was calculated by the following formula: Tumor volume [mm3] = (length[mm])×(width [mm])^2^ × 0.52.

### *In vivo* luciferase image and quantitative assay

Tumor implantation and treatment were the same as described above except that OT-I cells were initially transduced with a retroviral vector containing the luciferase gene (pRV-luc) before they were adoptively transferred to the animals. For *in vivo* imaging, mice were initially given an intraperitoneal injection of D-luciferin (150 mg/kg; PerkinElmer, Waltham, MA). Afterwards, tumors were imaged using the IVIS^®^ Spectrum *in vivo* imaging system (PerkinElmer, Waltham, MA). Images were analyzed and quantitated using Living Image version 4.2 software (PerkinElmer, Waltham, MA) and represented as total flux measurements in photons/second.

For ex vivo luciferase quantitative assay, tumors were weighted, placed in cryovials and immediately snapped frozen in liquid nitrogen. Equal amounts of frozen tumors were individually pulverized into a fine powder by hand grinding with a dry ice-chilled porcelain mortar and pestle. Frozen tumor powders were resuspended in 500 μl of 1X cell culture lysis reagent (Promega, Madison WI). Tumor homogenates underwent freeze-thaw cycles twice, and were then centrifuged at 12000 rpm for 10 min at 4°C. Fifty microliters of the supernatant tumor lysates were mixed with 50 μl of Bright-Glo™ Luciferase Assay System (Promega, Madison WI) and luciferase activity was assessed using a SpectraMax^®^ multi-mode microplate reader (Molecular Devices, Sunnyvale, CA). Results are reported as luciferase activity normalized to protein content.

### Tumor chemokine profile measurement

Panc02-H7-OVA tumor was established on the right flank of NSG mice by tumor cell implantation as described previously. Once tumors reached the approximate size of 8-10 mm in diameter, mice were given single intratumoral injection of either PBS or 1×10^7^ pfu of FusOn-H2. Forty-eight h later, tumors were collected, cut into small pieces and placed in lysis buffer (50mM Tris-HCl, pH 7.4; 0.6M NaCl; 0.2% Triton X-100; 0.1mM PMSF; 0.1mM pepstatin; 0.1mM aprotinin; 0.1mM NaF; 0.1mM NaOV_4_). Tumors were then macerated in glass Tenbroeck tissue grinders with approximately 20 strokes. Tumor homogenates were centrifuged at 12,000 rpm for 15 min at 4°C and the supernatants were collected. The total protein in the supernatants was quantified by the Bradford method. Equal amounts of protein samples were added to the plate of Multi-Analyte ELISArray kit (Qiagen, Valencia, CA), which detects a panel of 12 chemokines (RANTES, MCP-1, MIP-1a, MIP-1b, SDF-1, IP-10, MIG, Eotaxin, TARC, MDC, KC, and 6Ckine). The ELISA assay was performed according to the manufacturer's instructions and analyzed with a Spectra Max multi-mode microplate reader (Molecular Devices, Sunnyvale, CA).

### Flow cytometry analysis on the infiltrating T cell subsets and chemokine receptor expression on OT-I cells

For detecting chemokine receptor expression on splenocytes harvested from OT-I mice, the cells were cultured overnight in RPMI 1640 complete medium containing 300 U/ml of IL2 before they were doubly stained with anti-mouse CCR2, CXCR3 and CCR5 antibodies, in combination with either anti-CD4 or anti-CD8 antibodies, for 30 min at 4° C. Cells were then analyzed with BD FACSAria II (BD Biosciences, San Jose, CA).

To analyze the T-cell subsets of OT-I cells within tumor tissues, tumors were collected from the mice that had been used for the imaging experiment (after the termination of the experiment). The excised tumors were placed in dissociation buffer (100 U/ml collagenase type I and 100 μg/ml DNase in RPMI) for 30 min at 37 °C. The dissociated tumor tissues were then filtered through a 40 micron filter and washed 3X with PBS containing 2% fetal bovine serum (FBS) before they were loaded to Histopaque density gradient (Sigma-Aldrich, St. Louis, MO) and sedimented at 400 X *g* for 30 min. The interphase layer that was enriched with lymphocytes and monocytes were collected and counted. Cells were then stained with anti-mouse CD4 and CD8 antibodies alone or doubly stained for CD4-CD62L and CD8-CD62L for 30 min at 4° C, after which, they were incubated with anti-mouse CD16/32 antibody 30 min to block no-specific Fc receptor binding. The cell staining was analyzed with BD FACSAria II.

### Statistics

All quantitative data are reported as mean ± SD. Statistical analysis was made for multiple comparisons using analysis of variance and Student's *t*-test. P value < 0.05 was considered to be statistically significant.

## SUPPLEMENTARY MATERIAL AND FIGURE


