# Supplementary material for: An HSV-2 based oncolytic virus can function as an attractant to guide migration of adoptively transferred T cells to tumor sites
Source: Oncotarget. 2014 Nov 25;6(2):902–14. doi: 10.18632/oncotarget.2817 (PMC4359264; doi:10.18632/oncotarget.2817)

An HSV-2 based oncolytic virus can function as an attractant to guide migration of adoptively transferred T cells to tumor sites

Supplementary Material

Supplementary figure 1. Tumor growth of individual animals from data shown in Figure 6B.

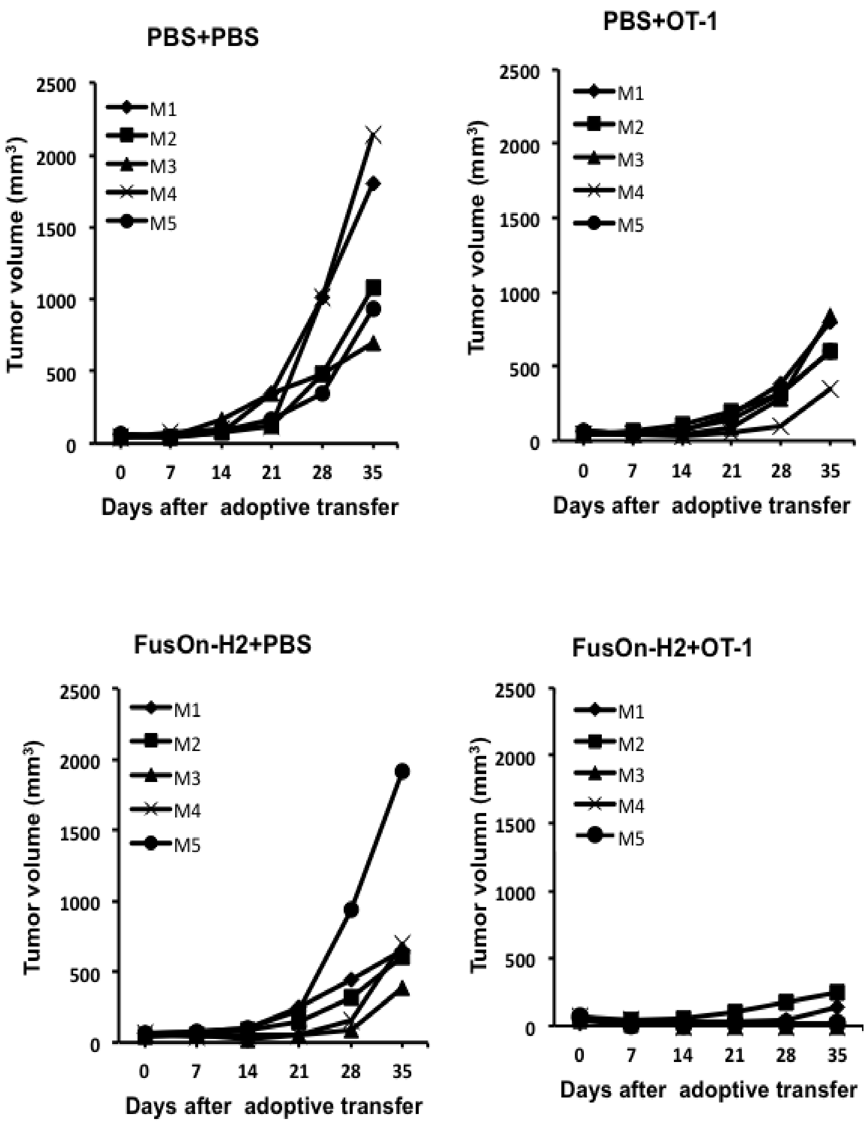

Supplement: Supplementary file 1 [file oncotarget-06-902-s001.pdf]
